# Choroidal neovascularization as a trigger for central serous chorioretinopathy

**DOI:** 10.1186/s40942-025-00761-7

**Published:** 2025-12-04

**Authors:** Aliénor Vienne-Jumeau, Elodie Bousquet, Jacques Bijon, Sarah Mrejen, Francine Behar-Cohen

**Affiliations:** 1 Centre Hospitalier National d’Ophtalmologie des Quinze-Vingts, Paris, France; 2 Ophtalmopôle, Cochin Hospital, Assistance Publique-Hôpitaux de Paris, Université Paris Cité, Paris, France; 3 Department of Ophthalmology, Lariboisière Hospital, Assistance Publique-Hôpitaux de Paris, Paris, France; 4 Department of Ophthalmology, Rothschild Foundation Hospital, 29 Rue Manin, Paris, 75019 France; 5 Ophthalmology Center for Imaging and Laser, Paris, France; 6 Centre de Recherche des Cordeliers, Université Paris Cité, INSERM U1138, Université Sorbonne Paris Cité, Paris, 75006 France; 7 Department Ophthalmology Hôpital Foch, Suresnes, France

**Keywords:** Choroidal neovascularization, Pachychoroid neovascularization, Central serous chorioretinopathy, Leakage point, Optical coherence tomography

## Abstract

**Background:**

To investigate whether choroidal neovascularization (CNV) may occasionally precipitate central serous chorioretinopathy (CSCR) in patients with pachychoroid features, by analyzing cases where fluorescein angiography (FA)-confirmed leakage originated directly within the CNV lesion.

**Methods:**

In this retrospective case series conducted at two tertiary referral centers, we reviewed patients with no prior history or signs of CSCR who presented with a first episode of CSCR and coexisting CNV between December 2024 and April 2025. Inclusion required at least one FA-confirmed leakage point located within the neovascular complex. Multimodal imaging—including FA, spectral-domain optical coherence tomography (SD-OCT), and optical coherence tomography angiography (OCTA)—was performed to detect CNV and evaluate its topographical relationship with leakage.

**Results:**

Among 202 patients screened, four met inclusion criteria (two males, two females; age range 54–58 years). All presented with a unilateral first episode of CSCR. In each case, FA and OCTA demonstrated precise colocalization of the leakage point within the CNV lesion. CNV was predominantly subfoveal and exhibited a mature morphology. Three patients were diagnosed simultaneously with CSCR and a neovascular membrane, while one developed CSCR during follow-up of a previously identified pachychoroid neovasculopathy. Subretinal fluid fluctuations were observed in all cases and often appeared independent of anti-VEGF treatment, suggesting a mechanism not exclusively driven by VEGF-mediated CNV activity.

**Conclusions:**

This case series suggests that, within the pachychoroid spectrum, CNV may not only complicate chronic or complex CSCR but may also act as a direct trigger of acute episodes. These findings underscore the importance of multimodal imaging, particularly FA and OCTA, for comprehensive assessment of CSCR. Given the small sample size and retrospective design, these observations should be interpreted as hypothesis-generating and warrant confirmation in larger prospective studies.

**Supplementary Information:**

The online version contains supplementary material available at 10.1186/s40942-025-00761-7.

## Introduction

Acute central serous chorioretinopathy (CSCR) is defined by the occurrence of serous retinal detachment associated with a leak on fluorescein angiography (FA), showing active dye passage through one or more areas of the retinal pigment epithelium (RPE) barrier break [1, 2] Other characteristic clinical signs are visual impairment such as contrast loss, color vision impairment, micropsia, and focal blurring, without major loss of visual acuity [3, 4] Spectral-Domain Optical Coherence Tomography (SD-OCT) examination confirms the diagnosis when characteristic choroidal signs are present, such as thickened choroid compared to fellow eye or age-matched controls and the presence of dilated veins that obscure the choriocapillaris (pachyvessels) [5, 6] and are associated with the serous detachment. On SD-OCT, the leakage site(s) observed on FA often correspond to structural changes such as a pigment epithelial detachment (PED), focal RPE disruption, areas of outer segment ‘erosion’ or elongation [3, 7], and, in some cases, adjacent hyporeflective subretinal lucencies [8]. In acute forms, FA typically reveals a focal site of RPE leakage. A high intensity of leakage is generally indicative of an episode that will resolve within a few months [5]. In contrast, diffuse RPE dysfunction without a clearly identified leakage site is more suggestive of chronic episodes and more complex disease forms [9]. The exact mechanisms of “leakage,” which is transient in acute forms, are not fully understood. Histological analysis of adrenaline-induced CSCR-like disease in monkey showed that spots of fluorescein leakage coincided with focal RPE cell degeneration over area of choriocapillaris endothelial defects with fibrin deposits within the Bruch’s membrane, suggesting that coagulation cascade and macromolecular transports could be involved [10]. Indocyanine green angiography (ICGA) in patients with acute CSCR confirmed choriocapillaris involvement with area of capillaries hypoperfusion and leakage at the site of RPE leak [11, 12]. Several mechanisms have been hypothesized, including bulk fluid flow [13] and reverse of retinal pigment epithelium pump [14], which could be promoted by mechanical stresses from the dilated and engorged choroid.

The development of choroidal neovascularization (CNV) is a sight threatening complication of CSCR, being responsible for a reduced visual acuity during a long-term follow-up [15]. Using multimodal imaging, CNV was detected in up to 40% of “chronic” or complex forms of CSCR [16]. The detection of CNV can indeed be difficult, and it is the presence of a flat irregular PED (FIPED), partially hyperreflective, that should raise suspicion [17, 18, 19] In CSCR, OCT angiography is currently considered the most sensitive modality for detecting CNV, outperforming FA and ICGA—particularly for type 1 CNV. In practice, OCTA enables visualization of CNV in approximately 35% of cases, compared with about 25% using ICGA. This is because, unlike in age-related macular degeneration (AMD), type 1 CNV in these conditions does not frequently form hyperfluorescent plaques in the late phase of ICGA [20]. Although FIPED have been identified in around 40% of acute CSCR, the exact occurrence of CNV in these cases has been poorly documented. Within the pachychoroid spectrum, another entity has been recognized: pachychoroid neovasculopathy (PNV). These are patients who have no history of CSCR but present mature neovascularization secondary to pachychoroid and area of ICGA hyperpermeability, with or without associated epitheliopathy and no exudative detachment [21]. Differential diagnosis with AMD can be difficult, and it is the thickness of the choroid and the absence of drusen that will guide this diagnosis, the boundary with AMD currently being debated [22]. 

In this ever-expanding spectrum of pachychoroid disease, the definition of different forms of CSCR is an ongoing process [23, 24] We report here the cases of patients presenting with a first episode of acute CSCR and an active FA leakage site located on the edge of a neovascular membrane. Based on these cases, we discuss the mechanisms, diagnostic methods, and implications for management.

## Methods

### Ethics statement

The study was approved by the Ethics Committee of the French Society of Ophthalmology (00008855). It adhered to the tenets of the Declaration of Helsinki (1964). As all patients were still under active follow-up, oral informed consent was obtained at the time of their next consultation to confirm their agreement for the use of anonymized clinical data and imaging.

### Study design

This retrospective observational study was conducted at the Department of Ophthalmology, Cochin Hospital (Paris), and the Department of Ophthalmology, Centre Hospitalier National d’Ophtalmologie des Quinze-Vingts (Paris). All patients aged ≥ 18 years who presented with either a new diagnosis or follow-up of CSCR associated with CNV between December 2024 and April 2025 were retrospectively reviewed for inclusion.

### Patients

Patients were eligible if diagnosed with acute CSCR associated with concurrent CNV. Inclusion criteria were: no prior history of CSCR, absence of clinical or imaging signs of complex CSCR, and presence of at least one leakage point located within the neovascular network. Multimodal imaging—including FA, ICGA, SD-OCT (Spectralis^®^, Heidelberg Engineering), and OCTA (Optovue^®^, Solix) —had to be performed within one month of CSCR diagnosis. Images with poor signal strength (OCTA quality index < 7/10 or significant media opacity affecting image interpretation were excluded. A prior history of symptomatic CSCR was defined as any previous visual symptoms suggestive of CSCR (such as transient metamorphopsia, micropsia, or blurred central vision), based on detailed chart review and patient interview. Prior asymptomatic or resolved episodes were excluded according to the following imaging criteria: (i) absence of RPE alterations (gravitational tracts, descending tracts, or mottled hypo-/hyperautofluorescence) on baseline or prior widefield fundus autofluorescence, with the absence of hyperautofluorescent areas indicative of a prior episode of subretinal fluid, as described by Freund et al. (JAMA Ophthalmology, 2013); (ii) absence of chronicity signs on spectral-domain optical coherence tomography (SD-OCT), including outer retinal or RPE atrophy (iii) no history of subretinal fluid (SRF) in previous imaging sessions.

### Data collection and analysis

For each patient, the following data were collected: age at diagnosis, gender, CNV pattern type, contralateral eye choroidal characteristics, and refractive error (spherical equivalent). The leakage point was defined as a focal area of hyperfluorescence with an increased in size over time on FA as previously described [25]. CNV was defined on OCTA as abnormal flow within the outer retinal slab [26]. When necessary, the outer retina and choriocapillaris slabs were manually adjusted to refine segmentation. CNV patterns were classified on en face OCTA projections (ORCC slab) according to the vascular morphology criteria established by Coscas et al. [27], distinguishing pattern 1 (typically lacy-wheel or sea-fan shaped) from pattern 2 (CNV not fulfilling all five criteria for pattern 1). Following identification of the leakage point and the CNV, OCTA and FA images were manually realigned by referencing retinal vessel bifurcation landmarks visible in both modalities at the level of the superficial microvasculature. The registration was performed by one observer (A.V.J.) and independently verified by a second observer (F.B.C.), who confirmed alignment accuracy. The mean registration error was estimated to be < 30 μm, based on residual vessel displacement across multiple reference points. Because all leakage points were located more than 50 μm from the image periphery and well within the central registered region, independent duplicate registrations were deemed unnecessary.

## Results

### Patients

Among 202 eligible patients with CSCR and CNV seen between December 2024 and April 2025, four patients met the inclusion criteria (Supplementary Fig. 1).

The cohort comprised two men and two women (Table 1). The age at CSCR onset ranged from 54 to 58 years. In all cases, only one eye was affected. The left eye was involved in two patients and the right eye in two. All patients were either emmetropic or mildly hyperopic, with absolute refractive errors < 3 diopters. At baseline, all patients were symptomatic at the onset of CSCR. The most frequently reported complaints were visual acuity (VA) loss and central scotoma. Specifically, Patients 1 and 4 presented with combined VA loss and scotoma, while Patient 3 experienced VA loss primarily attributed to cataract. Patient 2 reported a central scotoma without measurable VA decrease. Only one patient (Patient 2) had longitudinal imaging available before CSCR onset, with a five-year follow-up confirming the absence of prior SRF, while the remaining patients presented simultaneously with CNV and SRF at diagnosis. The subfoveal choroidal thickness (SFCT) of the affected eyes ranged from 258 μm to 420 μm at baseline, and SRF height from 0 μm to 284 μm. The fellow eyes showed SFCT values ranging from 230 μm to 492 μm, with no SRF detected.

**Table 1:** Description of included patients

	Gender	Eye	Dg	Age at CSCR dg	BCVA (Snellen)*	SFCT (µm)*	CVI*	SRF(µm)*	vessel type at dg	Vessel location	Number of leakage point(s)	Mid-phase HFP plaque (ICGA)	Late-phase HFP (ICGA)	Symptoms at CSCR dg	Time from symptoms to CSCR dg	Time from PNV to CSCR dg	Treatment	Follow-up duration (months)
Patient 1	Male	OS	PNV + CSCR	54	20/100 → 20/100**	398 → 377	0.67 → 0.68	96 → 59	Pattern 2	Subfoveal (temporal)	1	Yes	Yes	VA loss, scotoma	NA	At diagnosis	None	2
		OD	PNV with PVPD		20/20 → 20/20	374 → 376	-	-	-	-	-	-	-	No	-	-	-	
Patient 2	Female	OD	PNV + CSCR	54	20/20 → 20/20	391 → 267	0.58 → 0.57	0 → 127	Pattern 2	Subfoveal (infero-temporal)	1	Yes	Yes	Scotoma	0	12 months	16 IVT + topical DXM	126
		OS	FCE with PVPD		20/20 → 20/20	230 → 230	-	-	-	-	-	-	-	No	-	-	-	
Patient 3	Male	OD	PNV + CSCR	58	20/70 → 20/20	420 → 482	0.55 → 0.57	284 → 0	Pattern 2	Temporal	1	Yes	Yes	VA loss, attributed to cataract	None	At diagnosis	None	16
		OS	Uncomplicated pachychoroid with PVPD		20/20 → 20/20	492 → 498	-	-	-	-	-	-	-	No	-	-	-	
Patient 4	Female	OS	PNV + CSCR	54	20/200 → 20/100**	258 → 152	0.54 → 0.52	51 → 80	Pattern 2	Subfoveal (temporal)	1	Yes	Yes	VA loss	NA	At diagnosis	15 IVT	84
		OD	PPE with PVPD		20/20 → 20/20	317 → 278		-	-	-	-	-	-	No	-	-	-	

Structural and functional follow-up data are presented for both eyes, with the study eye listed first and the fellow eye below

* BCVA, SFCT, and CVI are presented at CSCR diagnosis and at the last follow-up

** Patient 1 had an unoperated cataract

Abbreviations: BCVA: Best corrected visual acuity; CSCR: Central serous chorioretinopathy; CVI: Choroidal vascularity index; Dg: Diagnosis; HFP: Hyperfluorescent plaque; PNV: Pachychoroid neovascularization; PPE: Pachychoroid pigment epitheliopathy; PVPD: pachyvessels and pachydrusens; SFCT: Subfoveal choroidal thickness; VA: Visual acuity

### CSCR diagnosis and diagnosis in the fellow eye

**Fig. 1:**
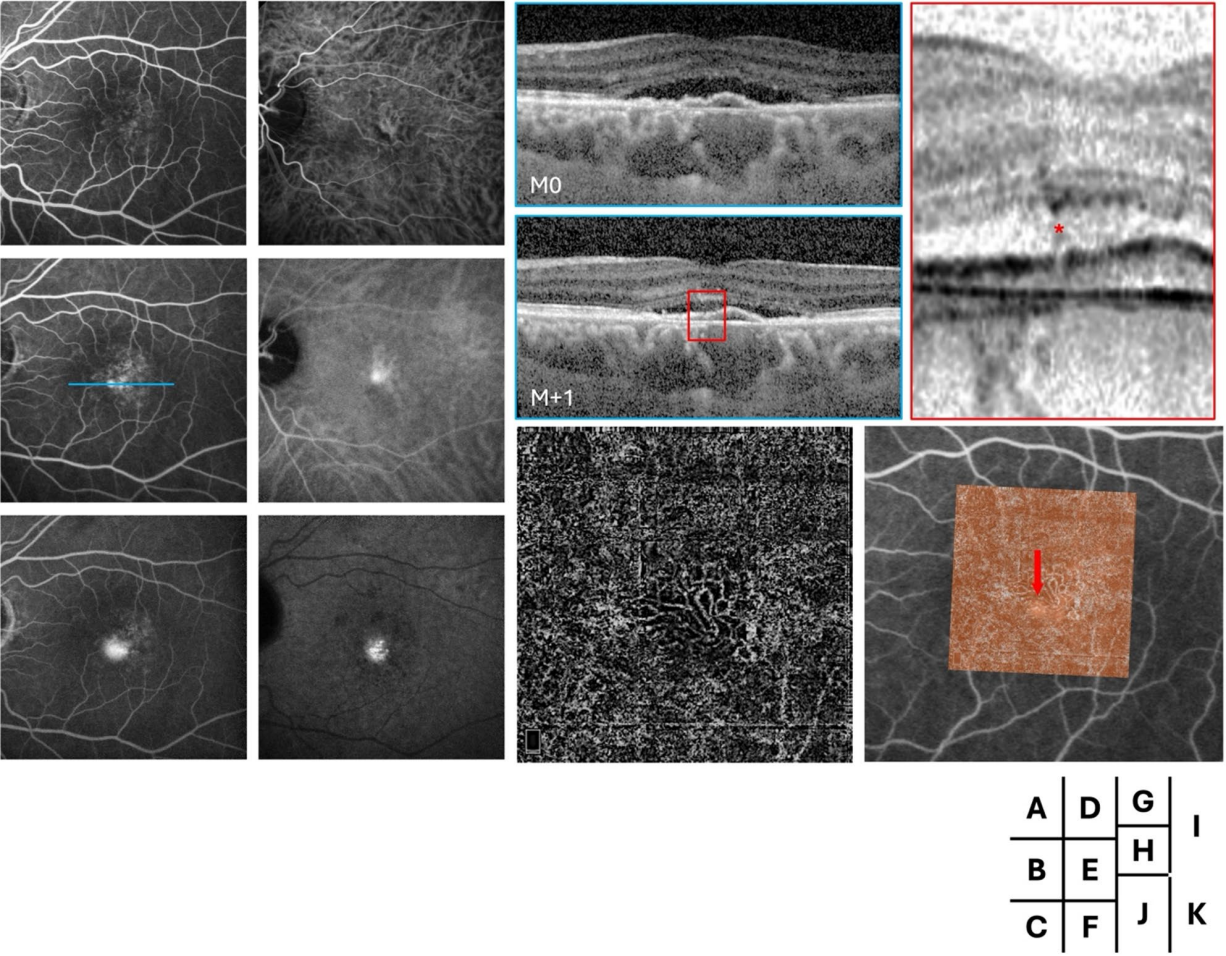
Multimodal follow-up imaging of the left eye in Case 1. Fluorescein angiography (FA) in the early, mid, and late phases (**A**–**C**) shows several hyperfluorescent pinpoint areas, including one with more intense leakage, while indocyanine green angiography (ICGA) in the early, mid, and late phases (**D**–**F**) reveals a parafoveal area of choroidal hyperfluorescence. Spectral-domain optical coherence tomography (SD-OCT) B-scans at the level of the leakage point (blue line) demonstrate subretinal fluid and a flat, irregular pigment epithelial detachment (FIPED) at baseline (**G**) and after several months of observation (**H**), with a magnified view highlighting a retinal pigment epithelium (RPE) break (red asterisk, **I**). OCT angiography (OCTA, **J**) and OCTA overlaid on FA (**K**) colocalize the choroidal neovascularization (CNV) with the leakage point (red arrow)

**Fig. 2:**
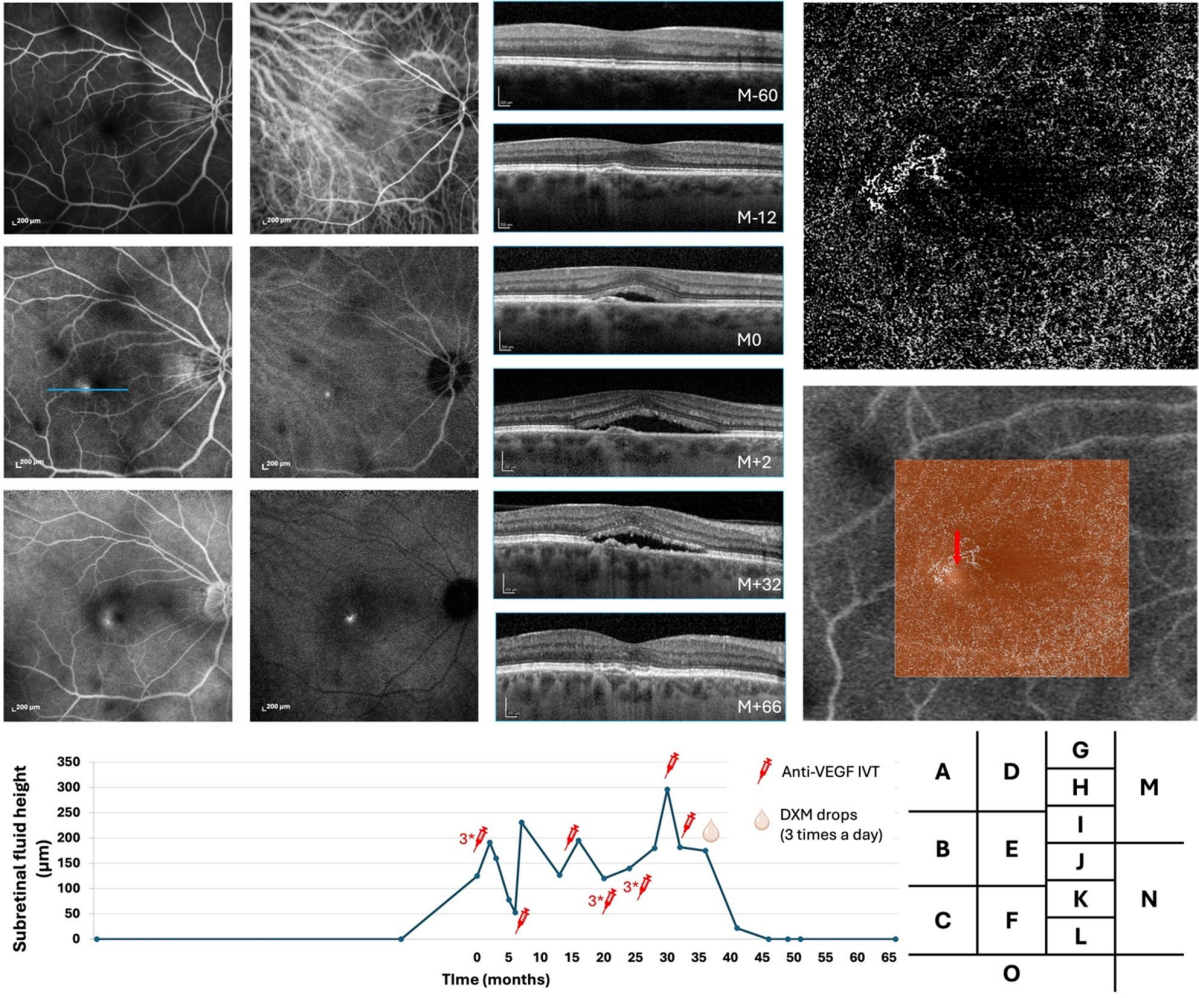
Multimodal follow-up imaging of the left eye in Case 2. Fluorescein angiography (FA) in the early, mid, and late phases (**A**–**C**) shows a subfoveal focal leakage, while indocyanine green angiography (ICGA) in the early, mid, and late phases (**D**–**F**) reveals prominent pachyvessels. (**G**–**L**) Serial spectral-domain optical coherence tomography (SD-OCT) B-scans through the leakage site (blue line in **B**) illustrate disease evolution : 60 months before baseline (**G**, M–60), choroidal thickening with a flat, irregular pigment epithelial detachment (FIPED) and no subretinal fluid; 12 months before baseline (**H**, M–12), early FIPED formation; at baseline (**I**, M0, day of angiography), new subretinal fluid and photoreceptor outer segment elongation; 2 months later (**J**, M + 2), increased fluid; 32 months later (**K**, M + 32), persistent fluid despite three intravitreal anti-VEGF injections; and 66 months later (**L**, M + 66), complete and durable resolution maintained without recurrence for three years. OCT angiography (OCTA, **M**) and OCTA overlaid on FA (**N**) demonstrate the colocalized choroidal neovascularization (CNV) and leakage point (red arrow). (**O**) Plot of SRF over time with treatment periods indicated. Topical dexamethasone (Dexafree^®^; three times daily) was initiated at month 36

**Fig. 3:**
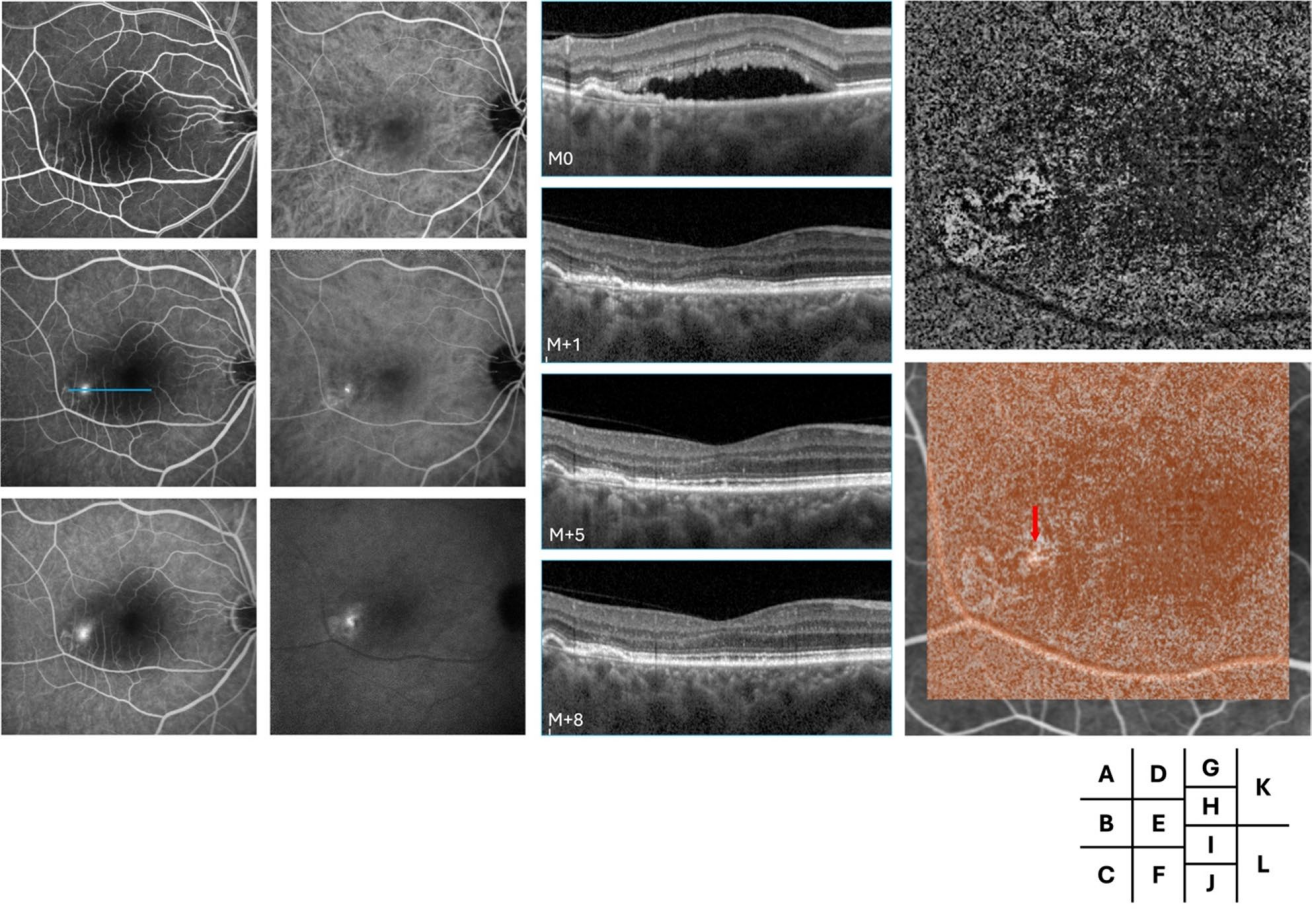
Multimodal follow-up imaging of the left eye in Case 3. Fluorescein angiography (FA) in the early, mid, and late phases (**A**–**C**) shows a focal hyperfluorescent leakage point, while indocyanine green angiography (ICGA) in the corresponding phases (**D**–**F**) reveals a parafoveal area of choroidal hyperfluorescence. Serial spectral-domain optical coherence tomography (SD-OCT) B-scans at the level of the leakage point (blue line, **G**–**J**) demonstrate subretinal fluid and a flat, irregular pigment epithelial detachment (FIPED) with associated fluid at presentation, which progressively spontaneously resolved over time. Optical coherence tomography angiography (OCTA, **K**) and OCTA overlaid on FA (**L**) colocalize the choroidal neovascularization (CNV) with the leakage point (red arrow)

**Fig. 4:**
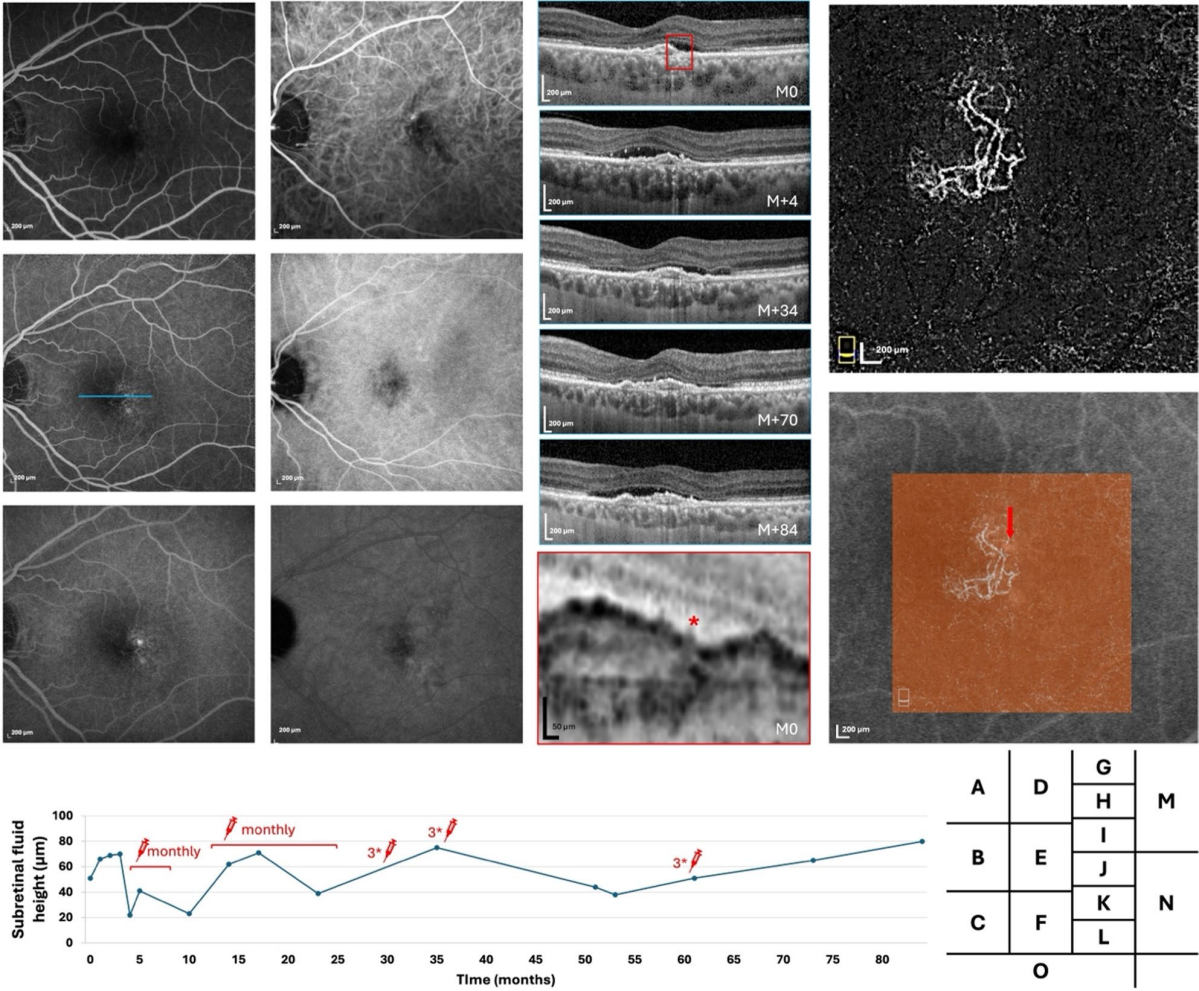
Multimodal follow-up imaging of the left eye in Case 4. Fluorescein angiography (FA) in the early, mid, and late phases (**A**–**C**) shows a subtle hyperfluorescent point, while indocyanine green angiography (ICGA) in the corresponding phases (**D**–**F**) reveals late hyperfluorescence at the leakage site. Spectral-domain optical coherence tomography (SD-OCT) B-scans at the level of the leakage point (blue line) demonstrate subretinal fluid and a flat, irregular pigment epithelial detachment (FIPED) at baseline (**G**) and after follow-up at 3 months, 2 years, 5 years, and 7 years (**H**–**K**), with a magnified view highlighting a retinal pigment epithelium (RPE) break (red asterisk, **L**). Optical coherence tomography angiography (OCTA, M) and OCTA overlaid on FA (**N**) colocalize the choroidal neovascularization (CNV) with the leakage point (red arrow). (**O**) Plot of SRF over time with treatment periods indicated

All cases presented with a first episode of CSCR, which by definition manifests as serous detachment associated with an angiographic leakage point in the context of pachychoroid. No signs of chronicity or complexity were observed, such as outer retinal atrophy or widespread and/or multifocal RPE atrophy (≥ 2 disc diameters) (Figs. 1A-F, 2A-D, 3A-D and 4A-D). The fellow eye of each patient showed distinct pachychoroid spectrum features. One patient had PNV, another had focal choroidal excavation (FCE), while the remaining two exhibited either pachychoroid pigment epitheliopathy (PPE) or uncomplicated pachychoroid. The patient with contralateral PNV manifest as a subtle flat irregular PED overlying a large and mature CNV and no sign or symptoms of an episode of subretinal fluid (Supplementary Fig. 2).

### CNV and leakage points

In all cases, the CNV lesions were located close to the fovea, with three classified as subfoveal (cases 1, 2, 4) and one as temporal (case 3). All CNV lesions in our series were classified as pattern 2 (intergrader agreement = 1.0).

Each patient exhibited a single fluorescein angiography-confirmed leakage point. In all cases, the leakage site colocalized precisely with the neovascular lesion identified on OCTA. Multimodal imaging for each case, including FA and OCTA overlay, demonstrated the clear spatial association between the area of FA-confirmed leakage and the corresponding CNV lesion. Other signs of leakage were observed on SD-OCT such as lucency in cases 1 and 3, outer segment erosion above RPE in case 3 and RPE break in cases 1 and 4 (Figs. 1I, 3 and 4L).

### Timing of CSCR onset and serous fluid fluctuations

In three patients, CSCR was diagnosed at the same time as the CNV. In the remaining case, CSCR developed 12 months after the initial identification of CNV in a patient with FCE.

Fluctuations in subretinal fluid were observed in all four cases, occurring independently of anti-VEGF IVT injections. These changes were not temporally associated with injections, suggesting a mechanism not solely driven by VEGF-dependent CNV exudative activity. In one case (Case 4), the subretinal fluid persisted despite monthly anti-VEGF injections (Fig. 4F–K).

## Discussion

This series of rare cases highlights a potential pathogenic link between CNV and the onset of acute CSCR in the context of pachychoroid. In these patients, FA revealed that the leakage point associated with and possibly responsible for subretinal fluid was located at the site of an RPE elevation above a CNV lesion. This spatial colocalization and the chronology of the events suggest that CNV may not merely be a late complication of chronic CSCR, but may instead act as a direct trigger for acute CSCR.

CNV has been typically recognized as a complication of complex/ chronic CSCR [28] in about 36% of cases and presenting as elevated RPE with double sign layer [29]. In a prospective longitudinal study, CNV complicated about 13% of CSCR, with higher risks for chronic cases, long-lasting fluid during the first year, older age and foveal involvement [30]. In the time course of chronic CSCR complicated by CNV, episodes of leakage can occur, often located in area of mid-phase ICG hyperpermeability, near or far from the neovessels and despite ongoing anti-VEGF treatment. It is therefore important to accurately locate the areas of leakage that could be treated by laser or focal PDT [31]. The multifactorial origin of the subretinal fluid in these cases could explain an incomplete response to anti-VEGF [32]. On the other hand, Pang and Freund described ten years ago, the PNV phenotype as the presence of type 1 CNV underlying a shallow RPE elevation in eyes with thick choroid and no evidence of exudative detachment or autofluorescence changes suggestive of previous or actual CSCR [21]. The angiographic characteristics of PNV are a late fluorescein leakage with undetermined origin, and a typical late staining plaque on ICGA [21]. The clinical presentation of our cases does not correspond to any of these descriptions and appears rather as a simple and acute form of CSCR whose leakage point overlooks a neovessel. Although not formally described, this clinical form was suggested, as neovessels could be added signs in simple or complex forms of CSCR when we proposed reclassifying the disease [33]. An important differential diagnosis for FA leakage overlying type 1 MNV in these eyes is exudation directly originating from the neovascular complex versus non-exudative leakage localized on top of the CNV. In our series, several features supported the latter: a single, well-defined leakage point on FA; a tent-shaped SRF configuration with photoreceptor outer segment elongation on OCT; and, in some cases, a fibrinous subretinal component associated with minute RPE discontinuities at the leakage site. The precise FA–OCTA registration, performed using superficial vascular landmarks with an estimated error < 30 μm, confirmed strict spatial colocalization of the leakage point with the CNV core, further supporting a non-exudative leakage mechanism localized on the neovascular membrane rather than diffuse exudation from active CNV.

The recognition of CNV-induced acute CSCR is important, as it may carry therapeutic implications and raise intriguing pathogenic hypotheses. From a diagnostic perspective, while SD-OCT is widely used in routine cases of acute or simple CSCR, it does not allow precise localization of leakage sites (as seen on FA) or direct visualization of CNV, which is better detected with OCT-A. However, the interpretation of OCT-A can be limited by signal attenuation in areas of subretinal fluid, making repeat imaging after fluid resolution often necessary. The best therapeutic option in this clinical presentation is uncertain and whether an initial combination of anti-VEGF and PDT would lead to more favourable outcome would need to be evaluated. PNV can be managed with either anti-VEGF or photodynamic therapy (PDT) [34]. In cases where PNV is associated with CSCR-like features, treatment response may differ. Specifically, CSCR-predominant PNV has been associated with more favourable outcomes, including a lower recurrence rate of subretinal fluid following PDT or anti-VEGF therapy [35]. In our cohort, several cases exhibited subretinal fluid fluctuations that appeared independent of anti-VEGF IVT. In such cases, targeted treatments—such as limited-spot PDT or possibly topical corticosteroids—may help reduce subretinal fluid and lower the risk of recurrence [36, 37]. In one patient, subretinal fluid resolved four months after initiation of topical dexamethasone three times daily. Although this temporal association does not imply causation; such observations nonetheless support further investigation into the potential modulatory effects of topical corticosteroids on choroidal and RPE function, as suggested by recent studies [38–41]. However, the efficacy and safety of these approaches in this specific context remain to be established and warrant evaluation in prospective clinical studies.

The mechanism underlying the leakage point in these cases is particularly interesting. It closely resembles the leakage point observed above a large choroidal vessel adjacent to Bruch’s membrane, where the choriocapillaris is no longer identifiable. While mechanical factors may play a role—given that mechanical stress activates the YAP/TAZ pathway [42], which is crucial for maintaining RPE differentiation [43] —they cannot be considered solely responsible. Serous retinal detachment (SRF) without a detectable leakage point is frequently observed in situations involving mechanical stress, such as dome-shaped macula, large drusenoid PED, or choroidal tumors. In these instances, diffuse RPE dysfunction is the underlying cause of SRF [44–46]. 

**Fig. 5:**
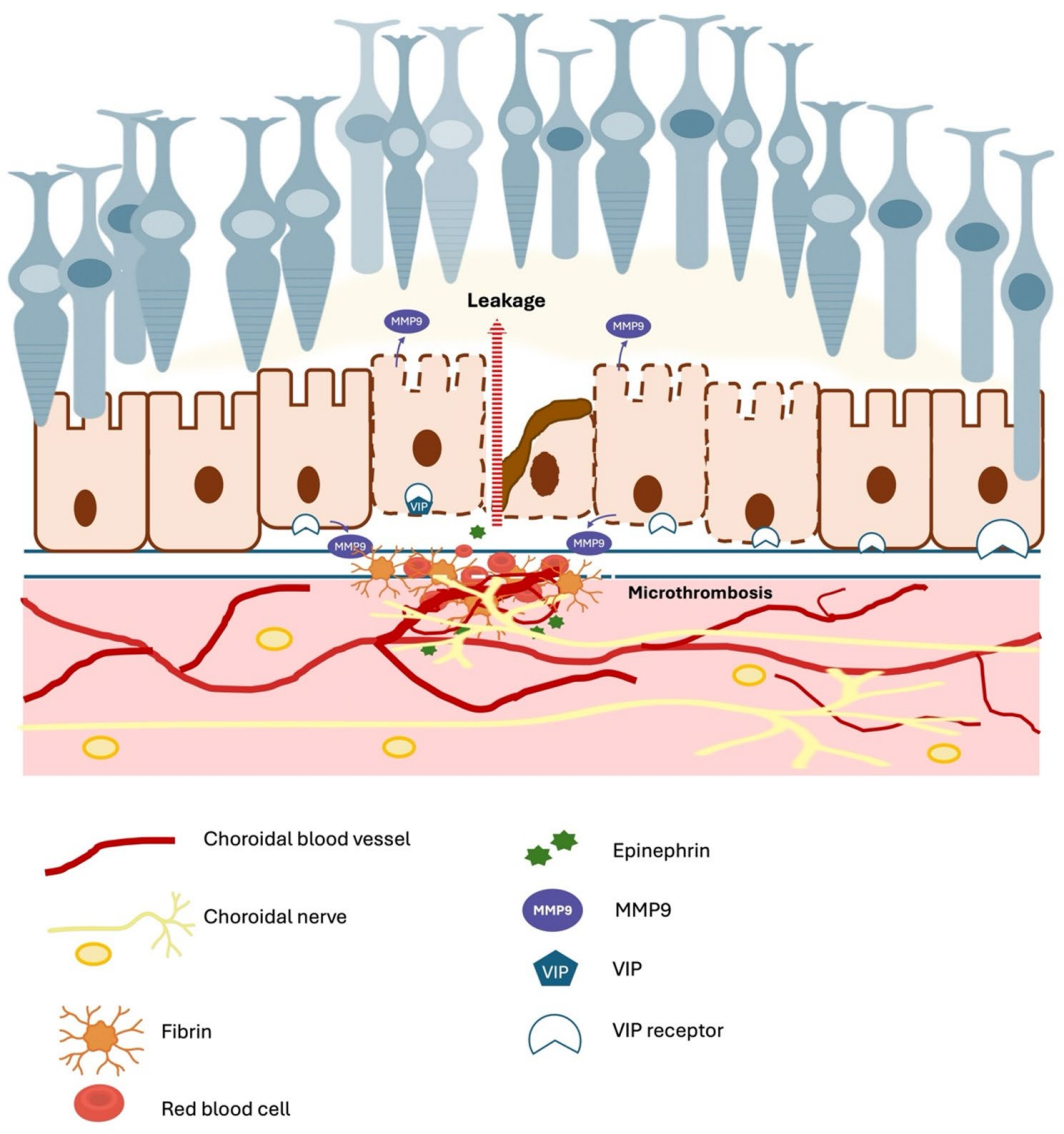
Schematic representation of the proposed hypothesis linking choroidal nerve signaling to leakage from choroidal neovascularization. Choroidal nerves release epinephrine, which activates matrix metalloproteinase-9 via VIP receptors on the retinal pigment epithelium (RPE). This disrupts RPE barrier integrity, facilitating fluid leakage. Simultaneously, choroidal microthrombosis contributes to local ischemia and inflammation, further promoting leakage

We hypothesize that, in pachychoroid disease, vascular remodelling is associated with—and may even be secondary to—choroidal autonomic neuropathy [47]. Under physiological conditions, the entire vascular system is regulated by the autonomic nervous system (ANS), and RPE homeostasis and transport activity are modulated by neuropeptides and adrenergic signalling. Large nerves and arteries are typically distant from the RPE, thereby preventing uncontrolled catecholamine discharges from activating the RPE, despite the presence of adrenergic receptors on its surface. In pachychoroid disease, pachyvessels with pathological innervation—similar to the mature neovessels often observed in CSCR [17]—may serve as a source of abnormal and uncontrolled neural activation due to their proximity to the RPE. From a pathophysiological perspective, dysfunctional vascular neural control may promote microthrombosis of the choriocapillaris, abnormal fibrin clotting, and RPE dysfunction, consistent with histological findings of RPE damage at leakage sites in monkeys [10, 48]. Indeed, choroidal haemostasis is under adrenergic control [49] and the sympathetic nervous system directly produces plasminogen activator around vessels [50]. Ion transport across RPE in under adrenergic signalling, and excessive epinephrine can induce RPE cell apoptosis [51]. On the other hand, vasointestinal peptide (VIP) controls RPE homeostasis and barrier function [52, 53]. Altogether, we hypothesize that choroidal neuropathy—potentially underlying the pachychoroid phenotype—combined with the close proximity of innervated vessels to the RPE, may contribute to RPE pump dysfunction, altered fibrinolysis, and matrix metalloproteinase activation, thereby possibly facilitating the development of CSCR. This proposed mechanism remains speculative but aligns with the concept of CSCR as a maladaptive stress response. A schematic representation of this hypothesis is shown in Fig. 5.

This study has limitations. The retrospective design and small sample size limit statistical generalizability. OCTA may be subject to projection artifacts and false positives, particularly in cases with poor fixation or significant subretinal fluid. The chronology of event could be challenged as non-symptomatic episodes might have occurred without notice. Indeed, no minimal duration of prior follow-up was required, as we considered this criterion excessively restrictive and potentially leading to the exclusion of relevant cases. In addition, we acknowledge that some patients were excluded due to incomplete multimodal imaging, which could have led to underrepresentation of cases where CSCR was well defined but the neovascular complex did not lie within the serous retinal detachment. Nevertheless, the consistent multimodal findings across cases support the concept that CNV may occasionally precipitate acute episodes of CSCR under certain conditions.

Ultimately, these findings suggest that CSCR may occasionally be associated with a neurovascular dysfunction initiated by quiescent CNV or choriocapillaris injury—challenging the traditional view of CNV as a consequence rather than a cause of CSCR.

## Supplementary Information

Below is the link to the electronic supplementary material.

Supplementary Material 1

## Data Availability

The datasets used and/or analysed during the current study are available from the corresponding author on reasonable request.
