# Supplementary material for: Choroidal neovascularization as a trigger for central serous chorioretinopathy
Source: Int J Retina Vitreous. 2025 Dec 4;12:4. doi: 10.1186/s40942-025-00761-7 (PMC12781755; doi:10.1186/s40942-025-00761-7)
Supplement: Supplementary file 1 — Supplementary Material 1 [file 40942_2025_761_MOESM1_ESM.docx]

**
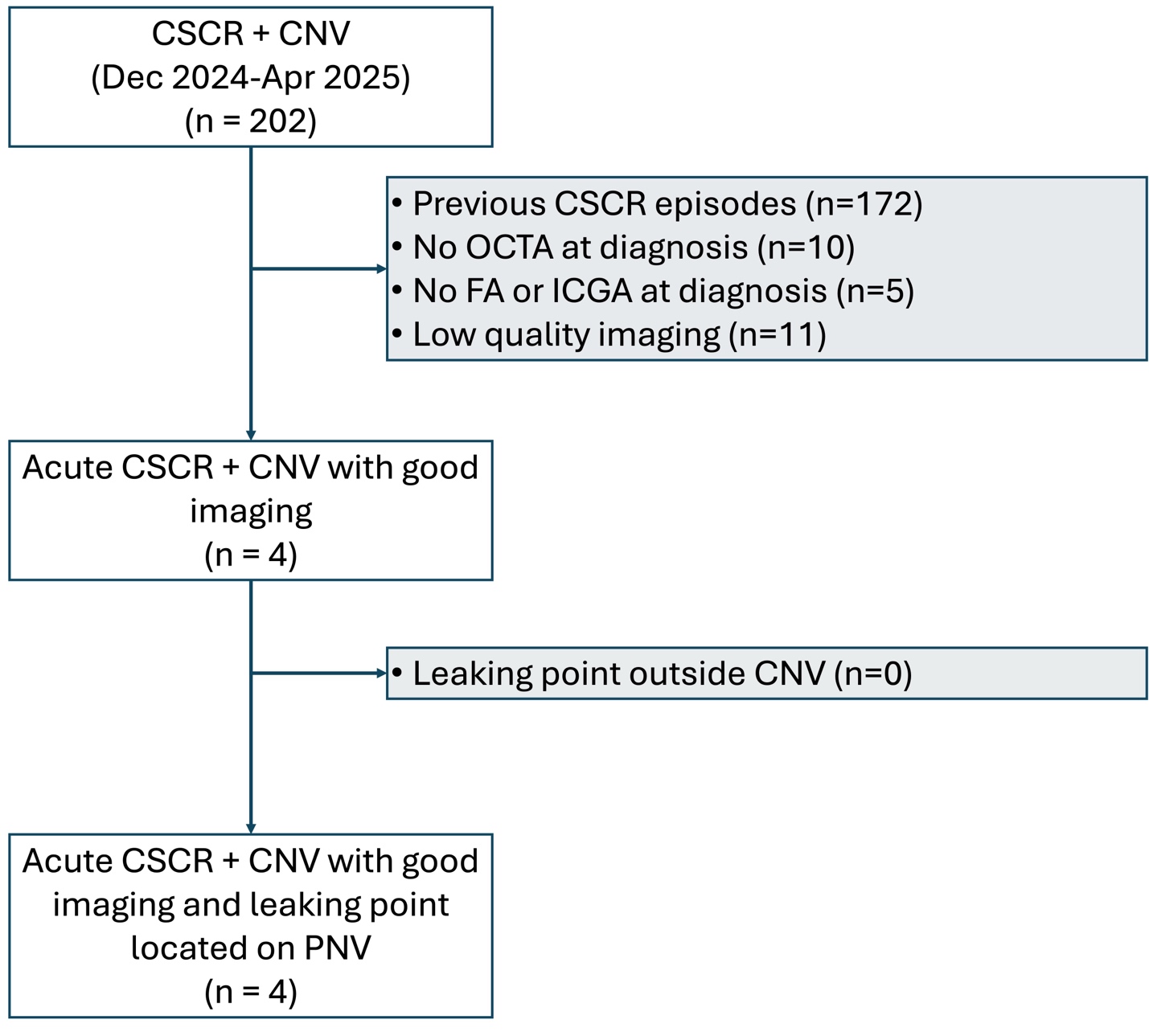
Supplementary Figure 1.** Flowchart of patient selection with CSCR and CNV (Dec 2024–Apr 2025). After exclusions, 4 cases of acute CSCR with CNV and good imaging were retained, all showing a leaking point on PNV.


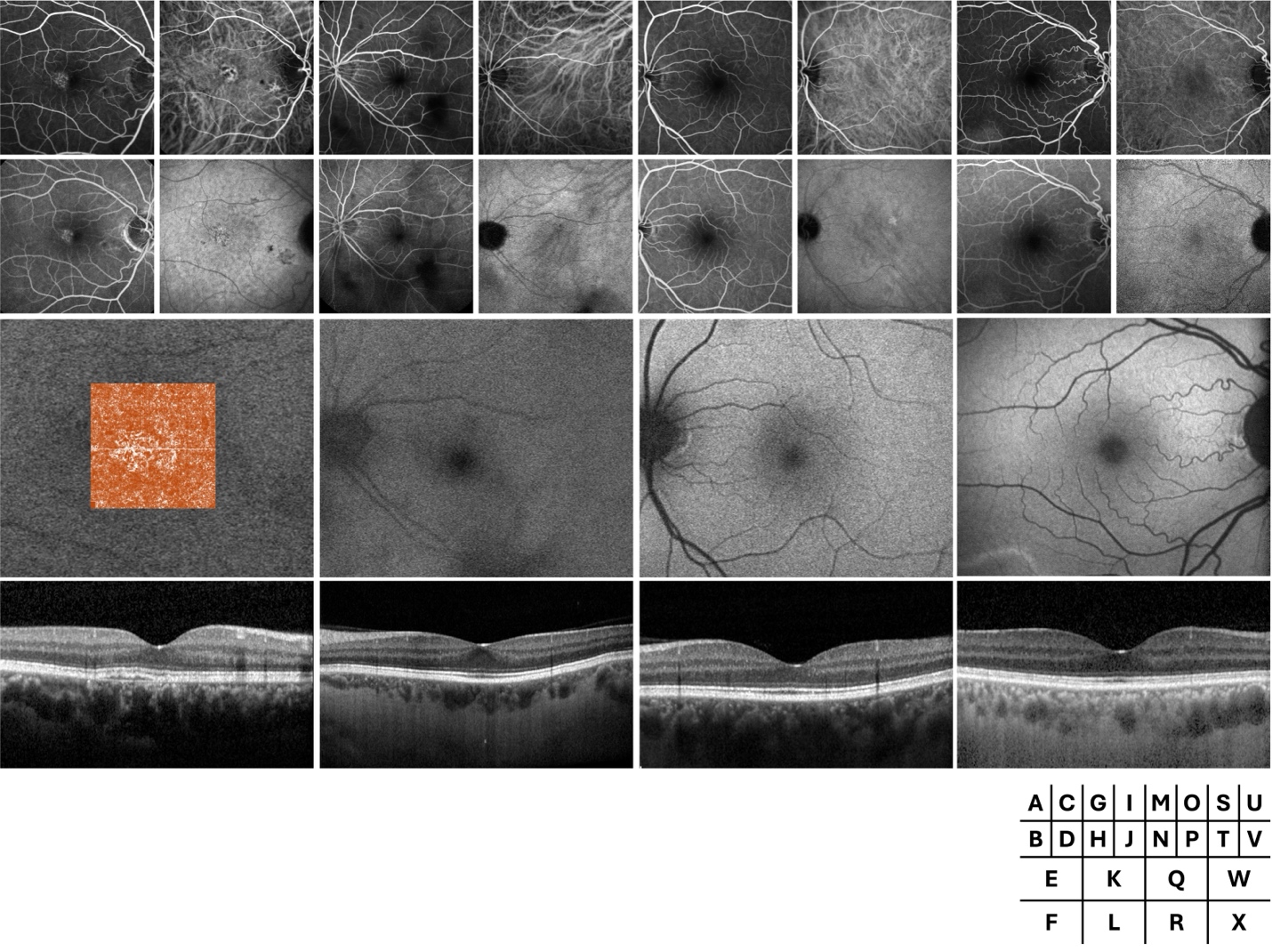


**Supplementary Figure 2.** Multimodal imaging of the contralateral eyes. For each patient (from left to right: patients 1 to 4), the following modalities are shown: early-phase fluorescein angiography (FA, top left), early-phase indocyanine green angiography (ICGA, top right), late-phase FA (middle left), late-phase ICGA (middle right), blue autofluorescence (BAF, center), and foveal optical coherence tomography (OCT, bottom). In Patient 1, a quiescent choroidal neovascularization (CNV) is visible, with optical coherence tomography angiography (OCTA) superimposed on the BAF image to better localize the lesion.
